# Impact of ADAR-induced editing of minor viral RNA populations on replication and transmission of SARS-CoV-2

**DOI:** 10.1073/pnas.2112663119

**Published:** 2022-01-21

**Authors:** Johan Ringlander, Joshua Fingal, Hanna Kann, Kasthuri Prakash, Gustaf Rydell, Maria Andersson, Anna Martner, Magnus Lindh, Peter Horal, Kristoffer Hellstrand, Michael Kann

**Affiliations:** ^a^Department of Infectious Diseases, Institute of Biomedicine at the Sahlgrenska Academy, University of Gothenburg and Sahlgrenska University Hospital, 41346 Gothenburg, Sweden;; ^b^Department of Microbiology and Immunology, Institute of Biomedicine, University of Gothenburg, 41346 Gothenburg, Sweden

**Keywords:** SARS-CoV-2, ADAR, RNA mutation, RNA deamination

## Abstract

Adenosine deaminases acting on RNA (ADAR) are RNA-editing enzymes that may restrict viral infection. We have utilized deep sequencing to determine adenosine to guanine (A→G) mutations, signifying ADAR activity, in clinical samples retrieved from 93 severe acute respiratory syndrome coronavirus 2 (SARS-CoV-2)–infected patients in the early phase of the COVID-19 pandemic. A→G mutations were detected in 0.035% (median) of RNA residues and were predominantly nonsynonymous. These mutations were rarely detected in the major viral population but were abundant in minor viral populations in which A→G was more prevalent than any other mutation (*P* < 0.001). The A→G substitutions accumulated in the spike protein gene at positions corresponding to amino acids 505 to 510 in the receptor binding motif and at amino acids 650 to 655. The frequency of A→G mutations in minor viral populations was significantly associated with low viral load (*P* < 0.001). We additionally analyzed A→G mutations in 288,247 SARS-CoV-2 major (consensus) sequences representing the dominant viral population. The A→G mutations observed in minor viral populations in the initial patient cohort were increasingly detected in European consensus sequences between March and June 2020 (*P* < 0.001) followed by a decline of these mutations in autumn and early winter (*P* < 0.001). We propose that ADAR-induced deamination of RNA is a significant source of mutated SARS-CoV-2 and hypothesize that the degree of RNA deamination may determine or reflect viral fitness and infectivity.

## Significance

Viral RNA may be edited by enzymes of the ADAR family that deaminate adenosine residues with ensuing A→G mutations. We found multiple A→G mutations in minor viral populations of the SARS-CoV-2 genome. A→G mutations accumulated in the receptor binding domain of the spike gene, which may cause structural changes by altering binding to the ACE2 receptor. Presence of A→G mutations in minor viral populations was associated with reduced viral load, implying that ADAR may limit viral replication. Analyses of >250,000 European samples from 2020 revealed that A→G mutations in SARS-CoV-2 RNA were inversely correlated with mortality as a reflection of incidence. ADAR may thus be important in providing new variants of SARS-CoV-2 with altered infectivity and transmissibility.

Double-stranded RNA (dsRNA) is formed during infection with positive-strand RNA viruses such as severe acute respiratory syndrome coronavirus 2 (SARS-CoV-2) and may be subject to editing by a group of enzymes denoted adenosine deaminase acting on RNA (ADAR) (1). The ADAR enzymes cause hypermutation by deaminating adenosine (A) residues to inosine (I) on dsRNA disrupting A to uracil base pairing (2). I is interpreted as guanosine during RNA replication and translation, and the A→I editing thus entails transition of A to guanosine (A→G). By this mechanism of RNA editing, ADAR enzymes may destabilize RNA and hence participate in first-line defense against RNA viruses. In addition, ADAR functions independently of RNA editing, including by protecting RNA transcripts from degradation (3, 4). In the respiratory tract, ADAR is preferentially expressed in endothelial cells of the upper airways with moderate expression in bronchial endothelium and lower expression in alveolar cells and lung macrophages (5).

There are three known mammalian forms of ADAR (ADAR1-3) (6). ADAR1 regulates the replication of several RNA viruses (7), but ADAR2 has hitherto only been reported to control infection of oligodendroglial cells with Borna disease virus (8). ADAR3 is absent outside of the central nervous system and is presumably not catalytically active. ADAR1 occurs in two isoforms, ADAR1p110 and ADAR1p150. ADAR1p110 is a nuclear enzyme, whereas ADAR1p150, which is interferon inducible, shuttles between the cytoplasm and the nucleus to allow editing of RNA viruses that replicate in the cytoplasm (9, 10). The notion that ADAR1 may limit replication of viral RNA is supported by results showing that genetic knockdown of ADAR1 in human liver cells markedly enhances the replicon of hepatitis C virus (11). However, ADAR1 activity may enhance replication of measles virus and influenza virus (12, 13). Altogether, these previous findings imply that ADAR-induced editing of RNA may contribute in viral evolution by providing virus variants with altered infectivity (14).

Mutations occur less commonly in the SARS-CoV-2 genome than in most other RNA viruses, but variants with altered replication efficiency have emerged (15, 16). The interest in the role of ADAR-induced editing of SARS-CoV-2 RNA was sparked by the detection of the D614G (A→G) mutation in the spike protein gene that may increase infectivity. Recent studies of SARS-CoV-2 consensus sequences, which reflect the dominant viral population in a sample, have identified low frequencies of A→G mutations (17–19). However, deep sequencing enables the detection of minor viral populations within samples. These minor viral populations may mirror the action of host factors, including ADAR, that edit viral RNA, and may also reflect variant virus development of relevance to infectivity.

This study utilized deep sequencing to analyze potential ADAR signatures (A→G mutations) in SARS-CoV-2 genomes retrieved from nasopharyngeal swabs from patients in the early phase of the COVID-19 pandemic. Four genomic segments were sequenced, including the RBD in the spike region and three segments that are targeted by diagnostic assays (RdRp, exonuclease, and E), covering 1,459 nt. In these samples, we detected minor viral populations of SARS-CoV-2 with potentially ADAR-edited RNA, and that the presence of these A→G mutations in the minor viral populations signified a lower viral load. We also analyzed 288,247 publicly available SARS-CoV-2 whole genome consensus sequences and observed that A→G mutations in the major viral population inversely coincided with the SARS-CoV-2 incidence. Our findings highlight that ADAR may be a potential source of mutation of SARS-CoV-2 RNA and point to the possibility that the emergence of ADAR-edited viral populations may influence virus fitness and infectivity.

## Results

### Patients and Virus Sequences.

Ninety-three samples were collected in March 2020 from 69 patients who were admitted to hospitals in the Västra Götaland region in Sweden and tested positive using SARS-CoV-2 PCR as part of routine diagnostics. Thirty-six patients had mild symptoms, 10 had moderate symptoms as defined by need of supplementary oxygen, 13 were admitted to ICU, and 10 patients died from the infection. Table 1 accounts for additional patient characteristics, with further details in *SI Appendix*, Table S1.

**Table 1.** Demographical and clinical parameters of patients

	Mild	Moderate	ICU*	Deceased	Overall	P^†^	Test
	(*n* = 36)	(*n* = 10)	(*n* = 13)	(*n* = 10)	(*n* = 69)		
Age, years							
Median	52	74.5	67	79	64	0.002	Kruskal–Wallis
IQR^‡^	41.8–66.0	48.5–80.8	54.0–72.0	73.25–86.8	49.0–78.0		
Sex							
Male (%)	16 (44.4)	5 (50.0)	10 (76.9)	7 (70.0)	38 (55.1)	0.16	Fisher's exact
Female (%)	20 (55.6)	5 (50.0)	3 (23.1)	3 (30.0)	31 (44.9)		
Fever, N							
Yes (%)	4 (40.0)	4 (57.1)	5 (83.3)	3 (75.0)	16 (59.3)	0.33	Fisher's exact
No (%)	6 (60.0)	3 (42.9)	1 (16.7)	1 (25.0)	11 (40.7)		
N/A^§^	26	3	7	6	42		
CRP, mg/L^¶^							
Median	33	110	160	240	103	0.002	Kruskal–Wallis
IQR^‡^	5.5–63.0	43.0–180.0	78.0–260.0	215.0–260.0	34.5–212.5		
N/A^§^	25	1	4	3	33		
Days from symptom onset to sampling							
Median	4	6.5	6	3	5	0.45	Kruskal–Wallis
IQR^‡^	3.3–6.5	2.3–10.0	5.0–9.3	3.0–5.0	3.0–8.5		
N/A^§^	22	4	7	5	38		
Comorbidity, N							
Yes (%)	8 (22.2)	4 (40.0)	6 (46.2)	3 (30.0)	21 (30.4)	0.38	Fisher's exact
No (%)	28 (77.8)	6 (60.0)	7 (53.8)	7 (70.0)	48 (69.6)		
Viral load (log_10_ genome copies/swab)							
Median	7.2	6.0	6.4	7.8	6.9	0.12	Kruskal–Wallis
IQR^‡^	5.3–8.6	5.6–8.5	5.2–6.8	6.9–8.8	5.5–8.5		

*ICU: admitted to intensive care unit.

^†^P: probability value of differences between groups. The applied tests are shown on the right.

^‡^IQR: Interquartile range.

^§^Not stated in patient medical records.

^¶^CRP value in patient plasma samples, indicating level of systemic inflammation.

All samples were deep sequenced and analyzed for minor and major viral population mutations. Eleven of the 93 samples had a consensus sequence (reflecting the major viral population) identical to the Wuhan strain. The nucleotide A23403G substitution (amino acid D614G) constituted the major viral strain in 70 of the remaining 82 samples. Apart from the D614G mutation, the number of A→G mutations in consensus sequences was consistently low (maximum: 4 nt; median: 1 nt).

**Fig. 1.**
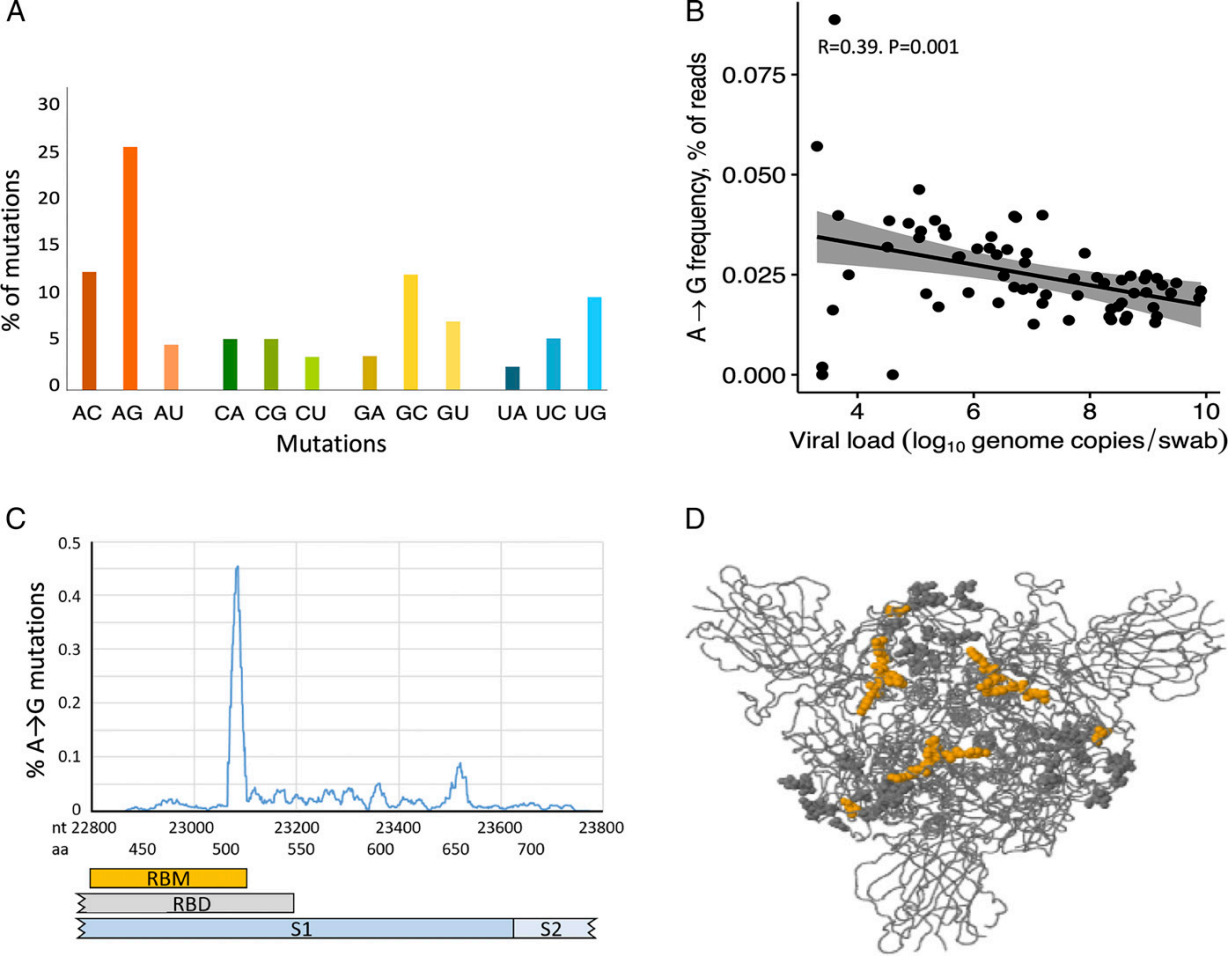
A→G mutations in minor viral populations of SARS-CoV-2. (*A*) Frequency of specific mutations in the SARS-CoV-2 genome. Results show the frequency of mutation, grouped by A, C, G, and U mutations, out of all mutations detected by Ion Torrent sequencing of the SARS-CoV-2 genome. The analysis was based on 98,325 changed residues that were detected from 5.04 × 10^9^ reads in 69 patient samples. Twenty-five percent of the changed residues were A→G mutations that constituted 0.035% of all reads. The difference between the proportion of A→G mutation and any other mutation was significant (*P* < 0.001, χ^2^ test). (*B*) Correlation between A→G mutations in minor viral populations of SARS-CoV-2 and viral load. Results show the viral load (log_10_ genome copies per swab) vs. A→G mutations in percent of all reads. Dots represent samples retrieved at first visit. Data were obtained by Ion Torrent sequencing in samples that passed sequencing quality thresholds and a viral load exceeding 4.5 log_10_ viral genome copies per mL. Viral load was analyzed by real-time PCR. (*C*) Distribution of A→G mutations in the spike region. Results show the A→G mutation frequency of minor viral populations within the sequenced part of the spike-encoding region of the SARS-CoV-2 genome. The *x* axis shows nucleotide positions and the corresponding amino acid residues of spike. The *y* axis shows the percentage of A→G mutations of adenosines merged mean mutational frequency over 20 nt. (*D*) Spike protein structure. The figure is a 3D visualization of the trimeric spike protein (21). Orange dots (amino acids G505C, G506R/S, Y508C, R509G, and Y523A) represent nucleotide A→G mutations associated with viral load in patient samples and amino acid changes in the RBM that were predicted to cause structural changes. Gray dots indicate A→G mutations with ensuing amino acid changes in the RBD that were not predicted to impact on the structure of RBD.

We analyzed relatively short sections of the SARS-CoV-2 genome (1,459 nt), which limited mutation detection in consensus sequences. However, by reducing the part of the genome sequenced, we were able to increase the sequencing depth to allow the detection of mutated minor viral populations. Analysis of samples by deep sequencing showed that, out of all sequence reads in all samples combined, a deamination RNA signature (A→G mutation) was observed in 0.035% of the coverage (depth) at positions with an adenosine residue. A→G substitution was the most frequent mutation in minor populations of SARS-CoV-2 (Fig. 1*A*; *P* < 0.001 for all comparisons). Of the 14,292 A→G mutations observed, 13,704 (96%) clustered with more than three mutations within 100 nt in all patients. A→G mutations were nonsynonymous in 47%.

To clarify whether the selection of sequenced nucleotides was representative for the entire SARS-CoV-2 genome, we performed full-length deep sequencing of nine samples from low-symptomatic individuals. *SI Appendix*, Fig. S1 shows the frequency of A→G mutations in all SARS-CoV-2 genes. The data allowed us to calculate that the A→G mutations in the 1,459-nt segment analyzed was 0.77-fold as frequent as the average of other parts of the SARS-CoV-2 genome.

### A→G RNA Mutations in Minor Viral Populations vs. Clinical Parameters.

The extent of A→G mutation in minor viral populations of SARS-CoV-2 RNA was not significantly associated with sex, comorbidity, fever, or time from onset of symptoms and did not differ between age groups (*SI Appendix*, Table S2). Also, there was no significantly different frequency of A→G mutation in samples recovered from patients with different COVID-19 severity. The frequency of A→G mutations in minor viral populations was significantly higher in patients with higher C-reactive protein (CRP; *P* = 0.02; *SI Appendix*, Table S2). The viral load at admission differed across disease stages (Table 1) and was independent of patient age (*SI Appendix*, Table S1*A*). Viral load did not significantly differ between disease stages (Table 1). As expected, patients with low viral load had a longer period between onset of symptoms and sampling (*P* = 0.007; *SI Appendix*, Table S1*D*). Twenty-four samples from 19 patients were analyzed to determine the appearance of A→G mutations in follow-up samples, showing a nonsignificant trend toward decrease of these mutations in minor viral populations over time (*SI Appendix*, Table S1*H*). However, we observed that 9 out of 10 follow-up samples from ICU patients showed an increase of A→G mutations at follow-up, which was not observed in other patient groups (*SI Appendix*, Fig. S2).

### A→G Mutations in Minor Populations of SARS-CoV-2 in Relation to Viral Load.

We aimed to define the potential impact of A→G mutations in minor viral populations on viral load and thus compared the frequency of A→G mutations in samples with higher or lower viral load, dichotomized by the median viral load at baseline (6.9 log_10_ genome copies per swab). We found A→G mutations at 387 positions and observed a significantly higher frequency of A→G mutations in samples with lower viral load (*P* < 0.001; Table 2). A similar reduction was observed in follow-up samples (*P* = 0.044; Table 2). Presence of A→C or A→U substitutions did not impact significantly on viral load (*SI Appendix*, Table S3). Causal diagram analysis by DAGitty did not identify confounding clinical parameters in analyses of A→G mutation vs. viral load (*SI Appendix*, Fig. S3).

**Table 2.** Association between A→G mutations on SARS-CoV-2 viral load

Viral load	N*	Ns^†^	A→G mutation frequency median (%)	P^‡^
At first sampling				
Less than median	34		0.031 (0.022–0.038)	<0.001
Greater than or equal to median	35		0.020 (0.016–0.024)	
At follow-up^§^				
Less than median		13	0.036 (0.026–0.047)	0.044
Greater than or equal to median		6	0.022 (0.019–0.027)	

*Number of patients and initial samples.

^†^Number of follow-up samples.

^‡^*P* value of Mann–Whitney *U* test regarding distribution of A→G mutation vs. viral load of samples.

^§^Viral load values at follow-up were stratified by the median viral load value of the baseline cohort.

The frequency of A→G mutations and the log viral load by correlation analysis showed a moderate negative correlation but with high significance (*P* = 0.001, R = −0.39; Fig. 1*B*).

Samples with <4.5 log_10_ genome copies per swab showed a higher variability of A→G mutations. At these low viral loads, less than 4,000 copies of SARS-CoV-2 RNA were present in the tested volume. Considering that we used a coverage of 4,000 reads as a minimum in the analysis, the low concentration thus leads to either overrepresentation or underrepresentation of mutated sites, which is reflected by the higher variability of A→G mutation frequency (Fig. 1*B*).

We performed a detailed analysis of A→G mutations in minor viral populations to determine the potential impact of each of the 387 observed A→G mutations on the viral load in the sample. At 83 out of 387 individual positions, the A→G mutation was significantly more frequently detected in samples with low viral load, and only one A→G substitution (a synonymous mutation at nucleotide 23512) occurred significantly more frequently in samples with high viral load (*SI Appendix*, Table S4).

### Genomic Distribution of A→G Mutations.

A→G mutations were found in all analyzed genes, that is, RdRp, exonuclease, spike, and envelope. However, we observed an accumulation of A→G mutations in two peaks in the spike region of SARS-CoV-2 (Fig. 1*C*). The first peak at nucleotide position 23076 to 23089 (amino acid positions 505 to 510) was in the receptor binding domain (RBD), and A→G mutations were detected in eight out of eight possible sites in 91% of the samples. The mutations changed four of the five amino acid residues YQPYR (amino acids 505 to 509) to CRPCG (Y505C, Q506R, Y508C, R509G; Fig. 1*D*, indicated in orange). The second peak localized in the C-terminal portion of S1 (nucleotide position 23512 to 23526; amino acids 650 to 655), and A→G mutations were found in six out of six possible sites in up to 76% of the samples. These mutations altered the amino acid sequence from LIGVEHV to any amino acid residue of LVGVGRV. We applied the CliqueSNV program (20) for analysis of minor viral populations and observed that none of the A→G mutations sites, including those detected in the receptor binding motif (RBM) region, coincided with another A→G-mutated site on individual viral genomes, indicating that A→G substitution occurred on separate RNA molecules.

### A→G Substitutions in Consensus Sequences of SARS-CoV-2 Strains Circulating during the COVID-19 Pandemic.

**Fig. 2.**
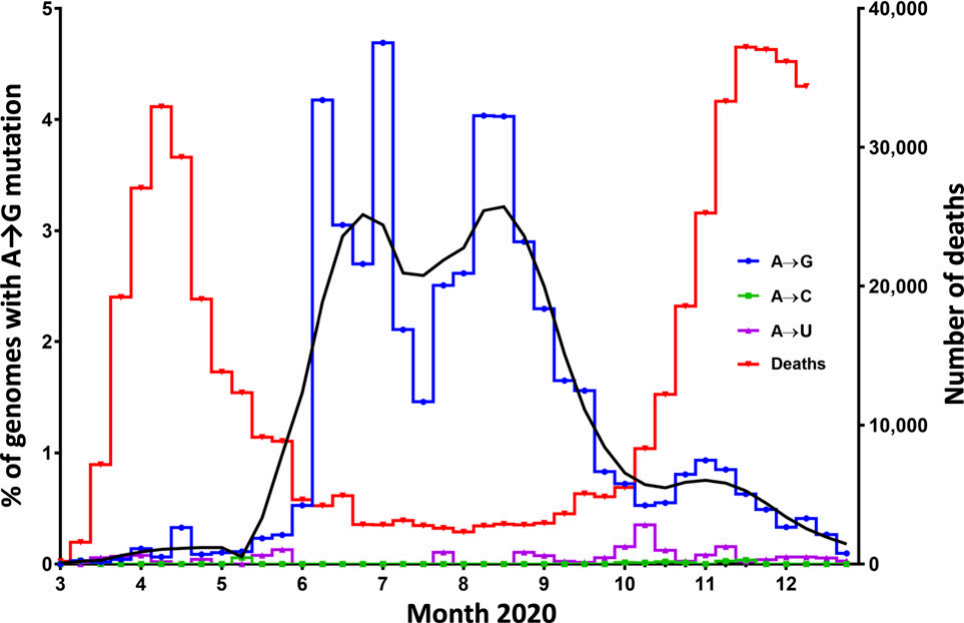
Prevalence of A→G mutations in SARS-CoV-2 genome residues vs. COVID-19 mortality in Europe. Consensus sequences of SARS-CoV-2 genomes from 186,616 European patients, sampled between March and December 2020, were retrieved from the GISAID database. The left *y* axis shows the percent of circulating genomes, and the right *y* axis shows the number of deaths. The blue line shows the frequency of SARS-CoV-2 genomes sampled at indicated time points (pooled weekly) that harbored at least one out of the 84 A→G mutated sites more commonly found in samples with low viral load in the previous deep sequence patient samples (*SI Appendix*, Table S4). The black line shows the same results by pooled 4-wk intervals. The magenta line shows A→C mutation-bearing SARS-CoV-2 genomes (weekly), and the green line shows A→U mutation-bearing genomes (weekly). The reduction of the frequency of A→G mutations between August and December was significant (*P* < 0.001, χ^2^ test). The red line shows the number of deaths caused by COVID-19 in Europe (European Centre for Disease Prevention and Control data collection https://www.ecdc.europa.eu/en/covid-19/data-collection) from March 1 to December 14, 2020), which was significantly associated with the frequency of A→G mutations (*P* = 0.004).

We next investigated whether A→G mutations that were observed in minor viral populations were detectable also in consensus (major) sequences in a public database Global Initiative on Sharing All Influenza Data (GISAID) (21). We analyzed 288,247 consensus sequences of which 186,616 were from Europe (samples obtained until December 31, 2020). We examined regions of the genome that were analyzed in our initial study of patient samples (RdRp, ORF1ab exocnuclease, spike, and E) and included downloaded genome sequences from all countries sharing data. Within Europe, the United Kingdom and Denmark provided the vast majority of sequences. In all SARS-CoV-2 genomes, the A→G mutations were more frequent than A→C or A→U mutations, indicating that deamination of RNA may be relevant to the evolution of SARS-CoV-2 (Fig. 2).

For Europe, we determined the frequency of A→G mutations in consensus sequences at any of the 84 positions that were found to be associated with viral load in minor viral populations in the initial patient cohort (as shown in *SI Appendix*, Table S4). We observed that the proportion of patients harboring any of these mutations (median one mutation, range one to two) in the consensus sequences increased gradually from a weekly median of 0.03% in March 2020 to 3.4% (*P* < 0.001) at the end of the first wave of COVID-19. With minor fluctuations, the abundance of SARS-CoV-2 carrying A→G mutations in the major viral population remained elevated throughout the summer when SARS-CoV-2 incidence, as reflected by COVID-19 mortality, was low. COVID-19 mortality was chosen as a proxy for incidence, as the latter depends upon the eligibility and accessibility of SARS-CoV-2 tests, which may have changed over time. We observed a decline of deaminated strains during the latter part of 2020, from 3.4% in August to 0.03% in December (Fig. 2). The abundance of A→G mutations in these consensus sequences showed a significant negative correlation with COVID-19-related mortality (Spearman rank order correlation test: *P* = 0.004, rho = −0.4577). A similar pattern of increased deamination during the summer of 2020 was observed in the United States (*SI Appendix*, Fig. S4), where, however, the spread of SARS-CoV-2 was more uneven as compared with Europe, and the number of analyzed sequences was lower.

More than 99% of the A→G mutations found in the European consensus sequences were associated with low viral load in the minor viral populations analyzed in our initial patient cohort. The most commonly deaminated sites were 18366 and 26319; of those, the former was prevalent during June to August, shifting to the latter during August until October. A majority of the deaminated strains belonged to three pango lineages, B.1, B.1.1, and B.1.258 (*SI Appendix*, Fig. S5) (22).

## Discussion

ADAR is a multifaceted aspect of RNA editing that comprises the deamination of A residues to I, which is transcribed to G during RNA replication. A first aim of this study was to determine the extent and localization of potentially ADAR-related gene signatures, that is, where A is replaced by G, in the SARS-CoV-2 genome along with attempts to define the potential impact of A→G mutations on viral load and on the course of disease.

In brief, we observed that samples collected in the early phase of the pandemic showed low frequency of A→G-mutated RNA residues in major (consensus) viral sequences, as reported by others (17, 23). However, residues with A→G substitutions were commonly detected in minor viral populations in which A→G was the most frequent mutation. An increased A→G mutation frequency was associated with low viral load and tended to increase during the course of infection in patients with severe disease and also increased with the degree of inflammation, as reflected by levels of CRP in blood.

A second aim was to determine the extent of A→G mutation in viral RNA by analysis of publicly available SARS-CoV-2 consensus sequences that were recovered during the first 9 mo of the pandemic (in 2020). Analysis of >250,000 genomes revealed an increase of A→G substitutions in SARS-CoV-2 consensus strains during March to April of 2020 that lasted throughout the summer months, followed by a decline during autumn and winter.

The observed maximal frequency of A→G mutation of 0.09% corresponds to ∼1.5 nt in the analyzed part of the genome (1,459 nt). Considering the representativeness of the A→G mutations in the analyzed segments, we estimated that ∼27 A→G RNA substitutions are present within each full-length SARS-CoV-2 genome.

Substantial evidence supports that the observed A→G mutations are caused by ADAR1-mediated deamination. A→G substitutions were more prevalent in patients with higher CRP, indicating accumulating deamination of RNA that may be secondary to inflammation. This finding agrees with the observation that RNA deamination is associated with induced expression of interferon-stimulated genes (24) and supports that the interferon-inducible ADAR1p150 rather than random mutations executes the observed accumulation of A→G mutations. Also, analysis of serial samples showed that the frequency of A→G mutations in minor viral populations tended to increase during infection among ICU-admitted patients, which is coherent with the pronounced inflammation characteristic of severe COVID-19 (25–27). The finding that A→G was the most frequent mutation in minor viral populations further supports that ADAR rather than error-prone virus replication is active in editing viral RNA. The latter finding was further supported by the finding that >95% of A→G mutations clustered in more than three mutations per 100 nt, which is a hallmark of ADAR1-induced deamination (28). These clusters were more than threefold more frequent than the ADAR deamination clusters observed in cellular RNAs in the absence of inflammation (28).

A→G substitutions in viral RNA in samples recovered from patients in the early phase of the COVID-19 pandemic were, with the exception of the D614G mutation, rare and largely confined to minor viral populations. However, SARS-CoV-2 variants with more-abundant A→G mutations gradually emerged in European consensus strains isolated during spring and summer of 2020, although to a limited extent. The finding of an association between A→G mutation abundance and low viral load in our original patient cohort agrees with the lower replication capacity previously observed for A→G mutated strains of the lymphocytic choriomeningitis virus (29).

We thus hypothesize that A→G mutated strains could only spread when a displacement by competing virus variants is low. Accordingly, strains carrying abundant A→G mutations may have become outnumbered at the onset of the second infection wave (16), although alternative mechanisms, including strain reversion, cannot be excluded. Interestingly, A→G substitutions in the SARS-CoV-2 genome in European consensus strains isolated during the summer of 2020 occurred almost exclusively at genomic sites that were associated with low viral load in the early-phase clinical samples.

Results achieved in our initial patient cohort identified several sites of A→G substitution in minor viral populations within the RBD of the spike gene, three of which were located within the RBM that interacts with the ACE2 receptor (30), although critical amino acids for ACE2-interaction are located upstream (amino acids 422 to 491) (31). Nonetheless, structural changes leading to altered ACE binding appear possible, as structural data from other groups show that the altered amino acids are in contact with other amino acids of S1 (32) and as this mutated segment is in the vicinity of mutations found in the variants B.1.351, B.1.1.28 (E484K), and B.1.1.7 (N501Y) (22). A second peak of A→G substitutions was observed between amino acids 650 to 655, which is within most immunogenic epitopes (amino acids 553 to 684) determined by others (33), which may thus alter virus transmissibility (16) or lead to escape from adaptive immunity (34). Our study may thus have identified additional potential sites of mutation that may alter viral infectivity and immunogenicity.

In conclusion, our findings imply 1) that A→G substitution in RNA residues, which likely reflects RNA deamination by ADAR, is the most commonly observed mutation in minor populations of SARS-CoV-2; 2) that these mutations are more commonly detected in patient samples with low viral load; and 3) that A→G mutations accumulate in the RBM region of S1. The finding that the prevalence of deaminated SARS-CoV-2 consensus strains shifted during phases of the COVID-19 pandemic merits further studies to clarify whether the kinetics of RNA deamination may determine infectivity and spread of SARS-CoV-2.

## Materials and Methods

### Samples and Patient Data.

Patients with PCR-verified SARS-CoV-2 infection with samples stored at the Sahlgrenska University Hospital Biobank from February 28 to March 31, 2020 were eligible for inclusion. Digital medical records were obtained from the central archive of Sahlgrenska University Hospital, Kungälv Hospital, and Norra Älvsborgs Länssjukhus and Uddevalla Hospital Group, Trollhättan, and accessed by the Melior program (Cerner). Patients were classified, according to disease severity, as mild (nonhospitalized or not receiving supplementary oxygen at hospital), moderate (hospitalized with supplementary oxygen), requiring admission to an intensive care unit (ICU), or dead from COVID-19. Comorbidities (present in 21 patients) included heart and coronary diseases, obesity, diabetes type 2, COPD, hypertension, and cancer. Fever was defined as a body temperature of ≥38 °C. There were 38 males and 31 females between the age of 1 and 97 y. Sixteen patients had fever at admission and 11 had a normal temperature, while, for 42 patients, these data were missing. CRP was documented for 36 patients (range 1 to 480 mg/L, median 103 mg/L), and the time between sampling and onset of symptoms was recorded for 31 patients (1 d to 16 d, median 4.5 d). In a subset of 24 samples, second and third samples were taken 1 d to 26 d after the first (median 4 d, average 5.5 d).

### SARS-CoV-2 PCR.

RNA was extracted from nasopharyngeal samples using a total nucleic acid extraction kit on the MagnaPure LC 2.0 instrument (Roche Life Sciences). Detection of SARS-CoV-2 RNA was performed using an in-house real-time PCR. Information about primers and reaction conditions are provided in *SI Appendix*, *SARS-CoV-2 RT-qPCR*.

### Amplification and Sequencing.

RNA was extracted from nasopharyngeal samples using a total nucleic acid extraction kit on the MagnaPure LC 2.0 instrument (Roche Life Sciences). RNA was reversely transcribed using the SuperScript IV complementary DNA (cDNA) synthesis kit (Thermo Fisher). PCR was performed on cDNA using fusion primers targeting regions within the RdRp, exonuclease, spike, and envelope regions of the SARS-CoV-2 genome (*SI Appendix*, Table S5). Fusion primers targeting RdRp, exonuclease, spike, and envelope of SARS-CoV-2 also contained adapters for Ion Torrent sequencing, and those were attached on amplicons in the same PCR. Barcoding PCR, to enable pooling of samples, was performed in the same reaction. Concentrations of barcoded DNA libraries from all samples were measured and adjusted to concentrations before pooling and Ion Torrent sequencing (35). Details are provided in *SI Appendix*, *Pre-NGS Amplification* and *Pooling and NGS*.

### Bioinformatics.

Reads were imported to CLC genomics workbench (Qiagen) as fastq files, and reads were trimmed. Adapter sequences and barcodes were trimmed off automatically in the Ion Torrent server program (Thermo Fisher). The CLC Genomics Workbench Trim Reads tool (Qiagen) was used with standard settings for removal of common tags, short reads, and low-quality reads (minimum read length was 65 nt, and reads not passing trimming were discarded before mapping). Reads were mapped to the SARS-CoV-2 reference genome (GenBank acquisition number NC_045512) using CLC Genomics Workbench 11 Map reads to reference tool (Qiagen). Reads mapping results were exported as tsv files containing data of nucleotide coverage at each position of the reference genome.

Reads mapped to the spike region were subject to in-depth single-nucleotide polymorphism (SNP) analysis and SNP haplotype calling using CliqueSNV, as previously described (20). Short reads settings were used, and all SNPs with a frequency of >0.05% of reads were considered. A→G changes belonging to the same haplotype of minor viral populations according to CliqueSNV were interpreted as an accumulation of A→G mutations in the viral subpopulation.

Structural protein analysis was made using CoVSurver: Mutations Analysis program on GISAID. Sequences based on the Wuhan reference containing all A→G changes that were found in the RBD region were uploaded as fasta files, and nonsynonymous changes were detected and assessed concerning impact on ACE2 affinity and antibody evasion.

### Deamination and Mutation Analysis.

Nucleotides were analyzed with >4,000 reads to ensure sufficient depth of sequencing sensitivity. Due to the technical limitations of Ion Torrent sequencing, we considered nucleotide changes only when abundant at ≥0.1% of the reads, which is twofold the technical error rate of this sequencing technique (35). These settings lead to underrepresentation of sites in samples in which less than 4,000 copies of SARS-CoV-2 genomes were present (>4.6 log_10_ genome copies per swab). Ct was translated to approximate viral load stated as log_10_ number of viral genome copies using the formula 14 − (Ct/3.3). ADAR1 deamination was assumed when A in the consensus sequence was mutated to G. To exclude non–ADAR1-mediated A→G mutations, which might be frequent at variable positions, we excluded positions in which A to C and A to T changes were more frequent. We further excluded A→G changes when occurring in >50% of the reads, assuming that G was present at the time of infection and not the result of ongoing disease.

### Statistics.

Within the baseline cohort (*n* = 69), we stratified continuous variables by their median values. This dataset was then merged with the follow-up dataset containing deamination estimates and viral load measurements. Subsequent grouping of the follow-up samples (*n* = 19) by their viral load values reflected their baseline viral load stratification status. Comparison of distributions across the independent groups of continuous variables was performed with Mann–Whitney *U* test (two groups) and Kruskal–Wallis test (more than two groups). In the analysis of 387 genomic positions with A→G mutation and their relation to viral load, Benjamini–Hochberg was used to adjust *P* values. Differences across paired samples (repeated measurements) were evaluated with the Wilcoxon signed-rank test. Differences between the frequencies of categorical variables were compared using χ^2^ (more than five counts per cell) or Fisher’s exact test (five or fewer counts in ≥20% of cells). Correlation between deamination and viral load or number of deaths was evaluated using the Spearman method.

Data handling, statistical analyses, and graphical representation were performed in R, version 4.0.3 (packages tidyverse, naniar, tableone and ggplot2) (36, 37) and in MS Excel. Distribution of A→G mutation and viral load of patient samples was visualized with function ggplot, method *“*loess*.”* Causal diagram analysis was performed with directed acyclic graph using tool for creating, editing, and analyzing causal diagrams [DAGitty, version 3.0 (38)].

### Mining of the GISAID Database.

A total of 288,247 complete SARS-CoV-2 genomes collected from humans until December 31, 2020 and submitted until February 2, 2021 were obtained from the GISAID database (available at https://gisaid.org) (39). The genomes were grouped by weeks of sampling (biweekly) and geographical location (Europe, North America, South America, Asia, Africa, or Oceania). Out of all of the sequences, 186,616 were from Europe. A custom Python script was used to extract the four regions of interest from all genomes and to determine the percentage of A→G, A→C, and A→T changes per adenosine site at these regions compared to the original Wuhan sequence. The percentage of genomes with nucleotide changes at sites carrying A→G sites were plotted using GraphPad Prism (v7.04).

### Ethical Statement.

The study was approved by the Swedish Ethical Review Board (application no. 2020-03276). Written informed consent was provided by all patients upon all sampling for SARS-CoV-2.

## Supplementary Material

Supplementary File

## Data Availability

Sequencing reads files (fastq files) for all patient samples were submitted to National Center for Biotechnology Information (NCBI) (BioProject accession PRJNA772935) and are accessible at https://www.ncbi.nlm.nih.gov/Traces/study/?acc=PRJNA772935 (40). Previously published data were used; consensus sequences obtained from GISAID are available at https://gisaid.org (39). All other study data are included in the article and/or *SI Appendix*.

## References

[1] S. Hur, Double-stranded RNA sensors and modulators in innate immunity. Annu. Rev. Immunol. 37, 349–375 (2019). 30673536 10.1146/annurev-immunol-042718-041356 PMC7136661

[2] U. Kim, Y. Wang, T. Sanford, Y. Zeng, K. Nishikura, Molecular cloning of cDNA for double-stranded RNA adenosine deaminase, a candidate enzyme for nuclear RNA editing. Proc. Natl. Acad. Sci. U.S.A. 91, 11457–11461 (1994). 7972084 10.1073/pnas.91.24.11457 PMC45250

[3] K. Licht, M. F. Jantsch, The other face of an editor: ADAR1 functions in editing-independent ways. BioEssays 39, 1700129 (2017). 10.1002/bies.201700129 28960389

[4] P. Deng , Adar RNA editing-dependent and -independent effects are required for brain and innate immune functions in *Drosophila*. Nat. Commun. 11, 1580 (2020). 32221286 10.1038/s41467-020-15435-1 PMC7101428

[5] M. Uhlén , Proteomics. Tissue-based map of the human proteome. Science 347, 1260419 (2015). 25613900 10.1126/science.1260419

[6] B. L. Bass , A standardized nomenclature for adenosine deaminases that act on RNA. RNA 3, 947–949 (1997). 9292492 PMC1369539

[7] C. E. Samuel, ADARs: Viruses and innate immunity. Curr. Top. Microbiol. Immunol. 353, 163–195 (2012). 21809195 10.1007/82_2011_148 PMC3867276

[8] M. Yanai , ADAR2 is involved in self and nonself recognition of Borna Disease Virus genomic RNA in the nucleus. J. Virol. 94, e01513-19 (2020). 31852792 10.1128/JVI.01513-19 PMC7158724

[9] J. B. Patterson, D. C. Thomis, S. L. Hans, C. E. Samuel, Mechanism of interferon action: Double-stranded RNA-specific adenosine deaminase from human cells is inducible by alpha and gamma interferons. Virology 210, 508–511 (1995). 7618288 10.1006/viro.1995.1370

[10] M. M. Lamers, B. G. van den Hoogen, B. L. Haagmans, ADAR1: “Editor-in-chief” of cytoplasmic innate immunity. Front. Immunol. 10, 1763 (2019). 31404141 10.3389/fimmu.2019.01763 PMC6669771

[11] D. R. Taylor, M. Puig, M. E. R. Darnell, K. Mihalik, S. M. Feinstone, New antiviral pathway that mediates hepatitis C virus replicon interferon sensitivity through ADAR1. J. Virol. 79, 6291–6298 (2005). 15858013 10.1128/JVI.79.10.6291-6298.2005 PMC1091666

[12] J.-F. Gélinas, G. Clerzius, E. Shaw, A. Gatignol, Enhancement of replication of RNA viruses by ADAR1 via RNA editing and inhibition of RNA-activated protein kinase. J. Virol. 85, 8460–8466 (2011). 21490091 10.1128/JVI.00240-11 PMC3165853

[13] R. Suspène , Double-stranded RNA adenosine deaminase ADAR-1-induced hypermutated genomes among inactivated seasonal influenza and live attenuated measles virus vaccines. J. Virol. 85, 2458–2462 (2011). 21159878 10.1128/JVI.02138-10 PMC3067779

[14] Z. J. Whitfield , Species-specific evolution of Ebola virus during replication in human and bat cells. Cell Rep. 32, 108028 (2020). 32814037 10.1016/j.celrep.2020.108028 PMC7434439

[15] B. Korber , Sheffield COVID-19 Genomics Group, Tracking changes in SARS-CoV-2 spike: Evidence that D614G increases infectivity of the COVID-19 virus. Cell 182, 812–827.e19 (2020). 32697968 10.1016/j.cell.2020.06.043 PMC7332439

[16] N. G. Davies , CMMID COVID-19 Working Group; COVID-19 Genomics UK (COG-UK) Consortium, Estimated transmissibility and impact of SARS-CoV-2 lineage B.1.1.7 in England. Science 372, eabg3055 (2021). 33658326 10.1126/science.abg3055 PMC8128288

[17] T. Mourier , Host-directed editing of the SARS-CoV-2 genome. Biochem. Biophys. Res. Commun. 538, 35–39 (2021). 33234239 10.1016/j.bbrc.2020.10.092 PMC7643664

[18] A. Graudenzi, D. Maspero, F. Angaroni, R. Piazza, D. Ramazzotti, Mutational signatures and heterogeneous host response revealed via large-scale characterization of SARS-CoV-2 genomic diversity. iScience 24, 102116 (2021). 33532709 10.1016/j.isci.2021.102116 PMC7842190

[19] M. Kosuge, E. Furusawa-Nishii, K. Ito, Y. Saito, K. Ogasawara, Point mutation bias in SARS-CoV-2 variants results in increased ability to stimulate inflammatory responses. Sci. Rep. 10, 17766–17769 (2020). 33082451 10.1038/s41598-020-74843-x PMC7575582

[20] S. Knyazev , Accurate assembly of minority viral haplotypes from next-generation sequencing through efficient noise reduction. Nucleic Acids Res. 49, e102 (2021). 34214168 10.1093/nar/gkab576 PMC8464054

[21] S. Elbe, G. Buckland-Merrett, Data, disease and diplomacy: GISAID’s innovative contribution to global health. Glob. Chall. 1, 33–46 (2017). 31565258 10.1002/gch2.1018 PMC6607375

[22] A. Rambaut , A dynamic nomenclature proposal for SARS-CoV-2 lineages to assist genomic epidemiology. Nat. Microbiol. 5, 1403–1407 (2020). 32669681 10.1038/s41564-020-0770-5 PMC7610519

[23] S. Di Giorgio, F. Martignano, M. G. Torcia, G. Mattiuz, S. G. Conticello, Evidence for host-dependent RNA editing in the transcriptome of SARS-CoV-2. Sci. Adv. 6, eabb5813 (2020). 32596474 10.1126/sciadv.abb5813 PMC7299625

[24] N. I. Vlachogiannis , Increased adenosine-to-inosine RNA editing in rheumatoid arthritis. J. Autoimmun. 106, 102329 (2020). 31493964 10.1016/j.jaut.2019.102329 PMC7479519

[25] X. Zhang , Viral and host factors related to the clinical outcome of COVID-19. Nature 583, 437–440 (2020). 32434211 10.1038/s41586-020-2355-0

[26] P. Mehta , HLH Across Speciality Collaboration, UK, COVID-19: Consider cytokine storm syndromes and immunosuppression. Lancet 395, 1033–1034 (2020). 32192578 10.1016/S0140-6736(20)30628-0 PMC7270045

[27] A. G. Laing , A dynamic COVID-19 immune signature includes associations with poor prognosis. Nat. Med. 26, 1623–1635 (2020). 32807934 10.1038/s41591-020-1038-6

[28] Z. Peng , Comprehensive analysis of RNA-Seq data reveals extensive RNA editing in a human transcriptome. Nat. Biotechnol. 30, 253–260 (2012). 22327324 10.1038/nbt.2122

[29] R. C. Zahn, I. Schelp, O. Utermöhlen, D. von Laer, A-to-G hypermutation in the genome of lymphocytic choriomeningitis virus. J. Virol. 81, 457–464 (2007). 17020943 10.1128/JVI.00067-06 PMC1797460

[30] J. Lan , Structure of the SARS-CoV-2 spike receptor-binding domain bound to the ACE2 receptor. Nature 581, 215–220 (2020). 32225176 10.1038/s41586-020-2180-5

[31] C. Yi , Key residues of the receptor binding motif in the spike protein of SARS-CoV-2 that interact with ACE2 and neutralizing antibodies. Cell. Mol. Immunol. 17, 621–630 (2020). 32415260 10.1038/s41423-020-0458-z PMC7227451

[32] R. Gowthaman , CoV3D: A database of high resolution coronavirus protein structures. Nucleic Acids Res. 49, D282–D287 (2021). 32890396 10.1093/nar/gkaa731 PMC7778948

[33] Y. Li , Linear epitope landscape of the SARS-CoV-2 Spike protein constructed from 1,051 COVID-19 patients. Cell Rep. 34, 108915 (2021). 33761319 10.1016/j.celrep.2021.108915 PMC7953450

[34] E. Callaway, Fast-spreading COVID variant can elude immune responses. Nature 589, 500–501 (2021). 33479534 10.1038/d41586-021-00121-z

[35] L. M. Bragg, G. Stone, M. K. Butler, P. Hugenholtz, G. W. Tyson, Shining a light on dark sequencing: Characterising errors in Ion Torrent PGM data. PLOS Comput. Biol. 9, e1003031 (2013). 23592973 10.1371/journal.pcbi.1003031 PMC3623719

[36] H. Wickham , Welcome to the Tidyverse. J. Open Source Softw. 4, 1686 (2019).

[37] T. J. Pollard, A. E. W. Johnson, J. D. Raffa, R. G. Mark, *tableone*: An open source Python package for producing summary statistics for research papers. JAMIA Open 1, 26–31 (2018). 31984317 10.1093/jamiaopen/ooy012 PMC6951995

[38] J. Textor, B. van der Zander, M. S. Gilthorpe, M. Liskiewicz, G. T. Ellison, Robust causal inference using directed acyclic graphs: The R package ‘dagitty.’ Int. J. Epidemiol. 45, 1887–1894 (2016). 28089956 10.1093/ije/dyw341

[39] GISAID, EpiCoV. https://www.epicov.org/epi3/frontend#310a61. Deposited 2 February 2021.

[40] J. Ringlander, PRJNA772935. BioProject. https://www.ncbi.nlm.nih.gov/Traces/study/?acc=PRJNA772935. Deposited 20 October 2021.

