# Supplementary material for: Impact of ADAR-induced editing of minor viral RNA populations on replication and transmission of SARS-CoV-2
Source: Proc Natl Acad Sci U S A. 2022 Jan 21;119(6):e2112663119. doi: 10.1073/pnas.2112663119 (PMC8833170; doi:10.1073/pnas.2112663119)
Supplement: Supplementary File [file pnas.2112663119.sapp.pdf]

Supplementary information for

Impact of ADAR-induced editing of minor viral RNA populations on replication and transmission of SARS-CoV-2

Johan Ringlander[1], Joshua Fingal[1], Hanna Kann[2], Kasthuri Prakash[1], Gustaf Rydell[1], Maria Andersson[1], Anna Martner[1], Magnus Lindh[1], Peter Horal[1], Kristoffer Hellstrand[1*] and Michael Kann[1]

[1]Department of Infectious Diseases, Institute of Biomedicine at the Sahlgrenska Academy, University of Gothenburg, Gothenburg, Sweden

[2]Department of Microbiology and Immunology, Institute of Biomedicine, University of Gothenburg, Sweden

*Corresponding author: Kristoffer Hellstrand

Email: kristoffer.hellstrand@microbio.gu.se

This PDF file includes:
Supplementary figures S1 to S5 (including legends)
Supplementary tables S1 to S5
Supplementary Methods and Material
SI References

Supplementary figures and tables

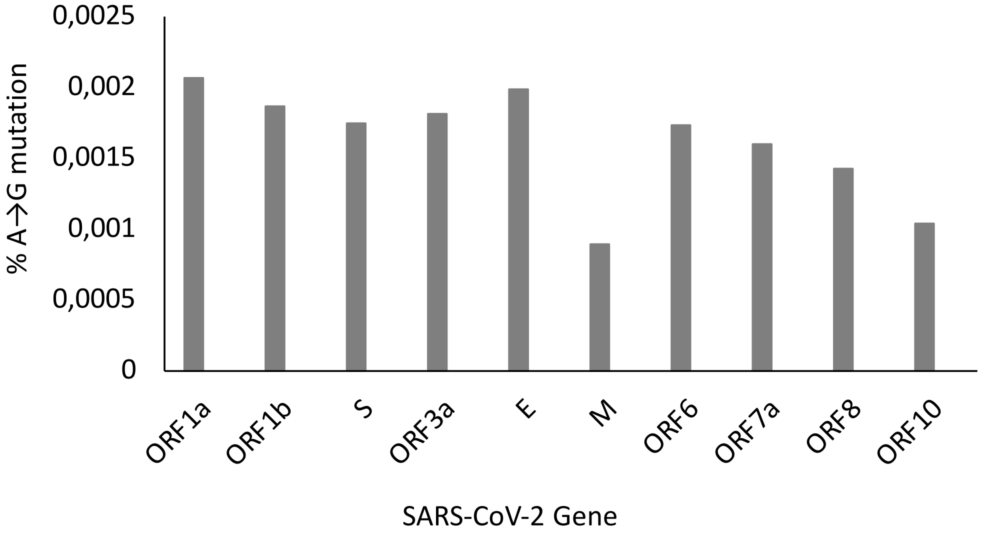

Supplementary Figure 1. A→G mutation in nine full-length deep sequences samples of low-symptomatic patients. X-axis: SARS-CoV-gene, y-axis: A→G mutation frequency in % of total A.

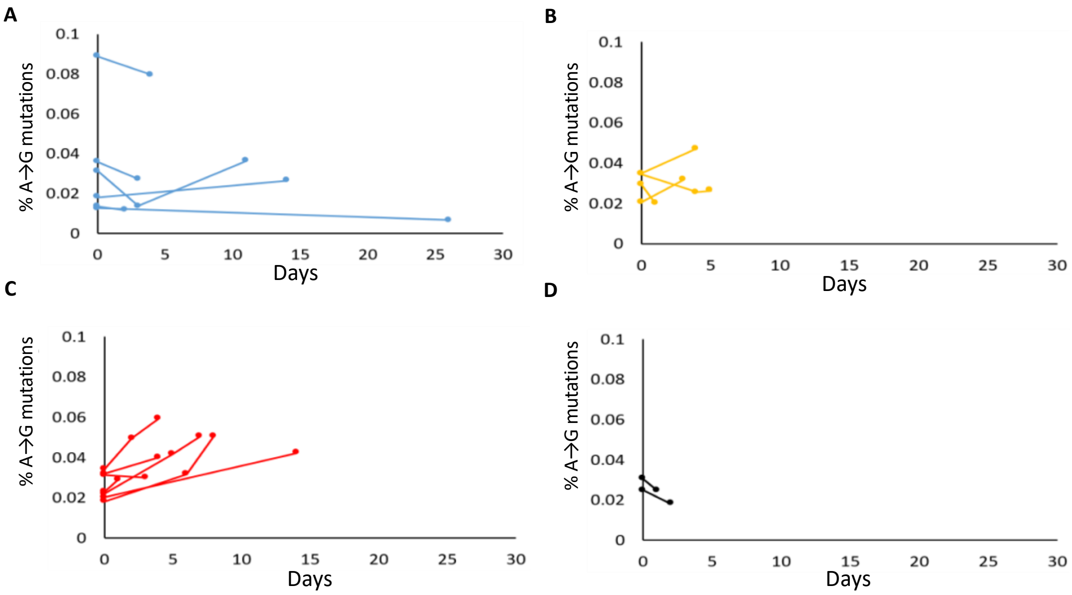

Supplementary Figure 2. Kinetics of A→G mutation in COVID-19 severity groups. A. mild. **B.** moderate. **C.** ICU. **D.** deceased. X-axis: time after first sampling in days. Y-axis: A→G frequency in % of total reads.

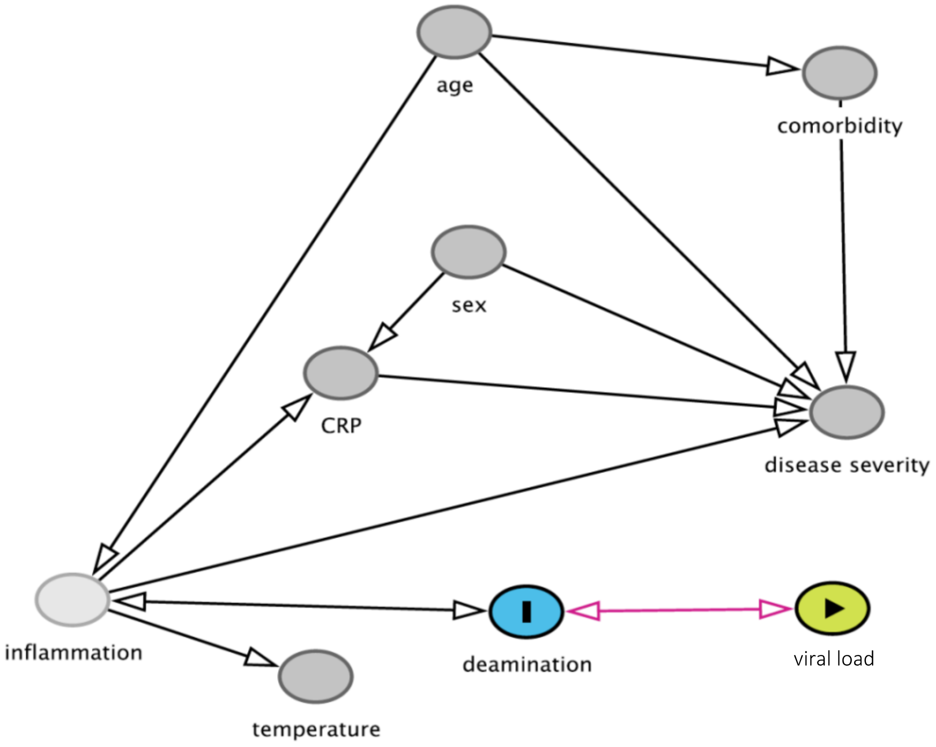

Supplementary Figure 3. Causal diagram analysis. The figure shows hypothesized interactions between measured and unmeasured parameters. Arrows: direct causal effects. Ellipses: green with a black triangle: exposure; blue with a black rectangle: outcome; dark grey: measured variable; light grey: unmeasured variable. The following causal links are hypothesized: age affects comorbidity and inflammation (1, 2). Co-morbidity, sex, age and inflammation affect COVID-19 severity (3, 4). Sex affects CRP (5), which is a proxy for systemic inflammation. No biasing path or confounder was identified within the given directed acyclic graph.

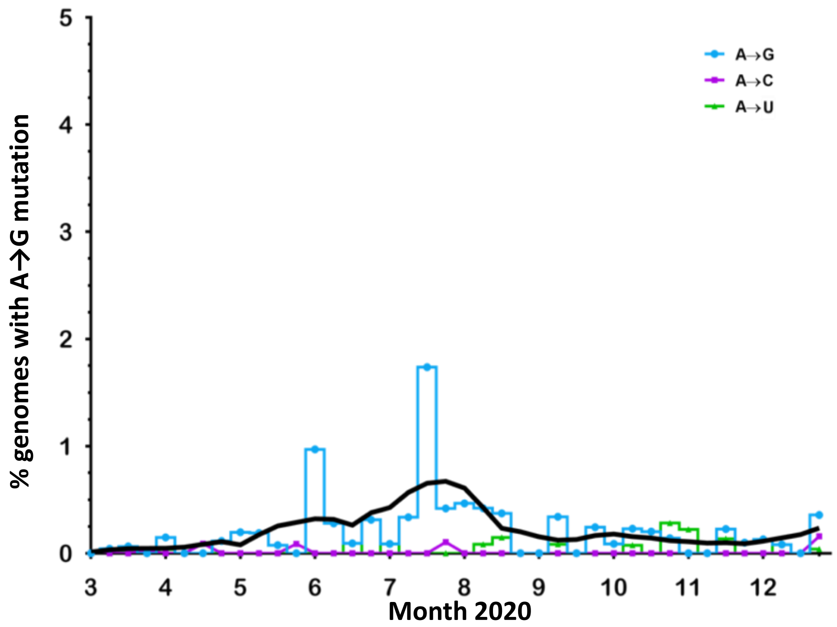

Supplementary Figure 4. A→G mutations in North American SARS-CoV-2 samples. Consensus sequences of SARS-CoV-2 genomes from 66,960 North American patients, sampled between March and December 2020 (retrieved from the GISAID database). Blue line, weekly: A→G mutation-bearing genomes (weekly). Black line: A→G mutation-bearing genomes (4-week-smooth). Magenta line, A→C mutation-bearing genomes (weekly). Green line: A→U mutation-bearing genomes (weekly). X-axis: month in 2020, y-axis: % of circulating genomes.

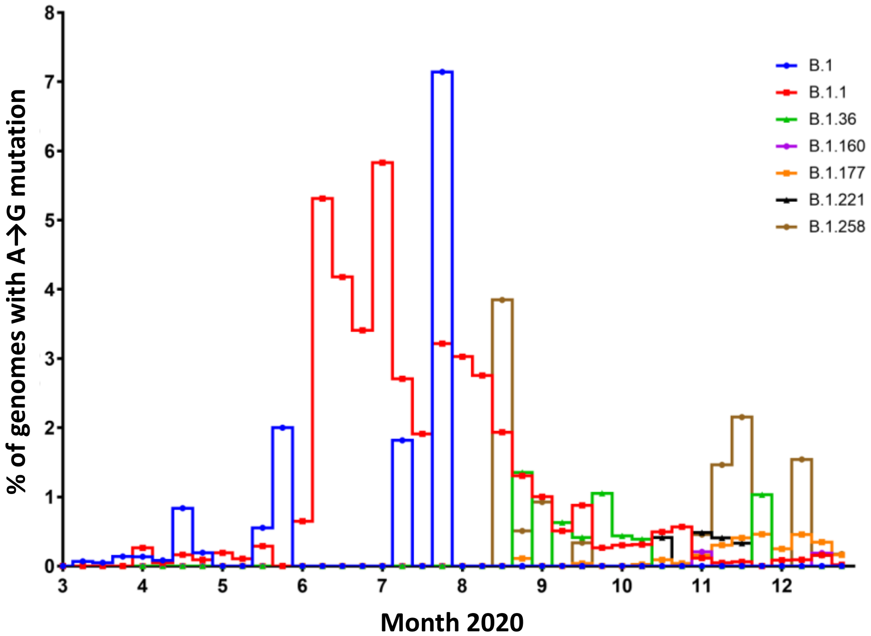

Supplementary Figure 5. A→G mutations in European Pango lineages of SARS-CoV-2. Pango lineages of sequences harbouring at least one of 84 A→G mutation sites, which were significantly negatively associated to SARS-CoV2 viral load, in strains circulating in Europe during 2020. X-axis: month in 2020, y-axis: % of circulating genomes.

Supplementary Table 1. Clinical parameters. Parameters are given on the top and on the left row of the table. Percentages are given in brackets. The statistical tests are indicated on the right.

A

	**Male (N=38)**	**Female (N=31)**	**Overall (N=69)**	**P**	**Test**
**Co-morbidity, N**					
Yes (%)	12 (31.6)	9 (29.0)	21 (30.4)	1	Chi^2
No (%)	26 (68.4)	22 (71.0)	48 (69.6)		
**Fever, N**					
Yes (%)	9 (60.0)	7 (58.3)	16 (59.3)	1	Fisher's exact
No (%)	6 (40.0)	5 (41.7)	11 (40.7)		
N/A[b]	23	19			
**Age, years**					
Median	63	64	64	0.731	Mann-Whitney U
IQR[a]	50.0 - 78.8	45.5 - 76.0	49.0 - 78.0		
**Viral load, log_{10} genome copies/swab**					
Median	6.6	7.7	6.9	0.239	Mann-Whitney U
IQR[a]	5.3 - 7.9	5.9 - 8.6	5.5 - 8.5		
**CRP, mg/L**					
Median	160	54	103	0.042	Mann-Whitney U
IQR[a]	78.0 - 240.0	14.9 - 155.0	34.5 - 212.5		
N/A	17	16			
**Time from symptom onset to sampling, days**					
Median	5	3	5	0.021	Mann-Whitney U
IQR[a]	4.0 - 10.0	2.3 - 4.8	3.0 - 8.5		
N/A[b]	21	17			

B

	**Co-morbidity**				
	**No (N=48)**	**Yes (N=21)**	**overall (N=69)**	**P**	**Test**
**Age, years**					
Median	56	79	64	0.005	Mann-Whitney U
IQR[a]	45.0 – 72.0	64.0 – 85.0	49.0 – 78.0		
**Viral load, log_{10} genome copies/swab**					
Median	7.0	6.8	6.9	0.974	Mann-Whitney U
IQR[a]	5.1 – 8.7	5.7 – 8.3	5.5 – 8.5		

C

	Time from symptom onset to sampling				
	<median (N=15)	≥median (N=16)	Overall (N=69)	P	Test
**CRP**					
Median	86	78	103	0.89	Mann-Whitney U
IQR[a]	20.0 – 210.0	43.0 – 150.0	34.5 – 212.5		
N/A[b]	6	3			
**Viral load, log_{10} genome copies/swab**					
N[c]	15	16			
Median	7.7	6.5	6.9	0.31	Mann-Whitney U
IQR[a]	5.5 - 8.9	5.5 - 6.8	5.5 - 8.5		

D

	Viral load				
	<median (N=34)	≥median (N=35)	overall (N=69)	P	Test
**Age**					
Median	62	64	64	0.46	Mann-Whitney U
IQR[a]	49.3 - 72.0	47.5 - 82.0	49.0 - 78.0		
**Days from symptom onset to sampling**					
Median	6	3	5	0.01	Mann-Whitney U
IQR[a]	4.0 - 10.0	3.0 - 5.0	3.0 - 8.5		
N/A[b]	16	22			

E

	C-reactive protein				
	< median (N=18)	≥ median (N=18)	Overall	P	Test
**Age**					
Median	69	68.5	64	0.72	Mann-Whitney U
IQR[a]	59.3 - 83.0	56.8 - 79.8	49.0 - 78.0		
**Viral load, log_{10} genome copies/swab**					
Median	7.0	6.4	6.9	0.11	Mann-Whitney U
IQR[a]	6.4 - 8.7	5.5 - 7.5	5.5 - 8.5		

F

	Fever				
	**No (N=11)**	**Yes (N=16)**	**Overall (N=69)**	**P**	**Test**
**Age, years**					
median	66	67	64	0.75	Mann-Whitney U
IQR[a]	51.5 - 83.5	54.5 - 81.5	49.0 - 78.0		
**Viral load, log_{10} genome copies/swab**					
Median	7.2	6.8	6.9	0.75	Mann-Whitney U
IQR	5.7 - 8.7	5.8 - 7.9	5.5 - 8.5		
**CRP, mg/L**					
Median	79	190	103	0.19	Mann-Whitney U
IQR[a]	38.3 - 157.5	75.0 - 225.0	34.5 - 212.5		
Missing data, N[c]	1	1			

G

	Sampling				
	**Initial**	**Follow-up**	**Overall**	**P**	**Test**
**Viral load, log_{10} genome copies/swab**					
N	19	19	38		
Median	6.4	4.5	6.0	0.01	Wilcoxon[c]
IQR[a]	5.3 - 8.1	3.7 - 7.3	4.4 - 7.5		

H

	A→G frequency				
	**Initial samples**	**Follow-up samples**	**Overall**	**P**	**Test**
**N[d]**	19	19	38		
Median	0.029	0.029	0.029	0.62	Wilcoxon[d]
IQR[a]	0.020 - 0.033	0.022 - 0.041	0.020 - 0.035		

[a]IQR: inter quartile range.
[b]N/A: not available.
[c]Number of samples.
[d]Wilcoxon signed rank test for paired samples.

Supplementary Table 2. A→G mutation frequency in patient groups.

		A→G frequency[a]			
	N[b]	Median	IQR[c]	P	Test
**Sex**					
Male	38	0.024	0.02 - 0.03		
Female	31	0.023	0.02 - 0.03	0.85	Mann-Whitney U
**Co-morbidity**					
No	48	0.023	0.02 - 0.03	0.57	Mann-Whitney U
Yes	21	0.023	0.02 - 0.03		
**Fever**					
Yes	16	0.027	0.012 - 0.03	0.37	Mann-Whitney U
No	11	0.030	0.02 - 0.03		
**Days from symptom onset**					
<median	15	0.024	0.02 - 0.03	0.87	Mann-Whitney U
≥median	16	0.024	0.02 - 0.03		
**Age**					
< median	34	0.023	0.02 - 0.03	0.71	Mann-Whitney U
≥ median	35	0.023	0.02 - 0.03		
**CRP, mg/L[d]**					
<median	18	0.021	0.02 - 0.03	0.02	Mann-Whitney U
≥median	18	0.031	0.03 - 0.03		
**Disease severity[e]**					
Mild	36	0.023	0.02 - 0.03		
Moderate	10	0.032	0.02 - 0.04	0.22	Kruskal-Wallis
ICU	13	0.021	0.02 - 0.02		
Deceased	10	0.025	0.02 - 0.03		

[a]Frequency of A→G mutations in minor viral populations among patient samples

[b]Number of patients

[c]Inter-quartile range

[d]C-reactive protein in patient plasma samples

[e]Mild: patient not hospitalized; Moderate: hospitalized with supplementary oxygen; ICU: treated at intensive care unit.

Supplementary Table 3. Viral load-associated non-A→G mutation frequencies. A. A→U mutation frequency in samples at admission. **B.** A→C mutation frequency in samples at admission.

A

	A→U mutation frequency in initial samples				
	**N**	**Median**	**IQR**	**P**	**Test**
Baseline viral load, log10 genome copies/swab					
<median	34	0.0083	0.0037 - 0.0385		
≥median	35	0.0035	0.0028 - 0.0222	0.06	Mann-Whitney U
overall	69	0.0051	0.0030 - 0.0322		

**B**	A→ C mutation frequency in initial samples				
	**N**	**Median**	**IQR**	**P**	**Test**
Baseline viral load, log10 genome copies/swab					
<median	34	0.0192	0.0110 - 0.0402		
≥median	35	0.0123	0.0074 - 0.0345	0.19	Mann-Whitney U
overall	69	0.0146	0.0090 - 0.0365		

Supplementary Table 4. Association between viral load and A→G mutations at defined positions in the SARS-CoV-2 genome.

Position[a]	P[b]	P adj.[c]	Change[d]	aa[e]	Position[a]	P[b]	P adj.[c]	Change[d]	aa[e]
15363	0.008	0.037	S		23303	<0.001	0.002	Non-s	Y581A
15367	0.008	0.037	Non-s	T5035E	23305	0.007	0.036	S	
15379	0.003	0.021	Non-s	S5039G	23324	0.004	0.021	Non-s	Y588A
18259	0.004	0.027	Non-s	I5999V	23354	<0.001	0.002	Non-s	I598V
18262	<0.001	0.003	Non-s	T6000C	23356	<0.001	0.001	Non-s	I598M
18273	<0.001	0.002	S		23357	<0.001	0.002	Non-s	Y599A
18282	0.008	0.037	S		23359	0.007	0.036	S	
18314	<0.001	0.002	Non-s	E6017G	23362	0.005	0.027	S	
18323	<0.001	0.005	Non-s	H6020R	23365	<0.001	0.007	S	
18328	0.008	0.037	Non-s	T6022A	23397	<0.001	0.025	Non-s	Y612C
18336	<0.001	<0.001	S		23400	<0.001	0.005	Non-s	G613R
18366	0.01	0.046	S		23418	0.011	0.05	Non-s	
18376	<0.001	0.005		T6038A	23419	0.005	0.027	Non-s	
23076	<0.001	<0.001	Non-s [f,g]	Y505C	23436	<0.001	0.009	Non-s	H625R
23079	0.00	0.028	Non-s [f,g]	G506R	23446	0.003	0.021	S	
23080	<0.001	0.005	non-s[f,g]	G506S	23476	0.007	0.036	S	
23083	<0.001	<0.001	S		23495	0.003	0.021	Non-s	T645A
23085	<0.001	0.008	Non-s [f,g]	Y508C	23503	<0.001	<0.001	S	
23087	<0.001	<0.001	Non-s [f,g]	R509G	23512[h]	0.002	0.014	S	
23089	<0.001	0.002	S		23535	<0.001	0.003	Non-s	N658S
23116	<0.001	0.009	S		23554	0.005	0.028	Non-s	I654M
23129	<0.001	0.007	Non-s [g]	Y523A	23588	0.005	0.027	Non-s	T676A
23140	<0.001	0.002	S		23614	0.001	0.012	S	
23156	0.007	0.036	Non-s	N532D	23623	<0.001	0.002	s	
23169	0.009	0.044	Non-s	N536S	23627	0.005	0.027	Non-s	S689G
23201	<0.001	0.009	Non-s	Y547A	23646	0.003	0.021	Non-s	Y695C
23203	<0.001	0.002	S		23656	<0.001	<0.001	S	
23207	0.001	0.013	Non-s	Y549A	23729	0.008	0.037	Non-s	T723A
23209	<0.001	0.003	S		23732	<0.001	0.008	Non-s	T724A
23223	<0.001	0.002	Non-s	E554G	26245	0.004	0.027	Non-s	M1?
23229	0.002	0.016	Non-s	N556S	26264	0.003	0.021	Non-s	E7G
23251	<0.001	0.007	S		26265	0.004	0.024	Non-s	
23253	0.002	0.016	Non-s	Q564N	26267	0.002	0.014	Non-s	E8G
23261	<0.001	0.001	Non-s	R567G	26271	0.003	0.021	S	
23263	0.002	0.014	S		26281	0.002	0.017	Non-s	I13V
23265	<0.001	0.002	Non-s	D568G	26290	<0.001	0.004	Non-s	S16G
23274	<0.001	0.002	Non-s	D571G	26319	0.005	0.027	S	
23276	<0.001	0.009	Non-s	T572A	26328	0.004	0.025	S	
23283	0.003	0.019	Non-s	D574G	26332	<0.001	0.002	Non-s	T30A
23295	0.002	0.017	Non-s	D578G	26334	<0.001	0.005	S	
23299	<0.001	0.002	S		26337	0.002	0.015	S	
23301	<0.001	0.002	Non-s	Q580R	26347	0.007	0.036	Non-s	T35A

[a]Only positions with a coverage frequency of >0.1% and a false discovery rate of 0.05 were considered. Numbering according to the Wuhan reference strain. The nt were identified by Mann-Whitney U tests comparing the A→G mutation frequency in baseline and follow-up samples (N=93) that passed sequencing quality thresholds. Samples were stratified by the median viral load (6.9 log10 genome copies/swab) of the initial samples (N=69).

[b]P-values by Mann-Whitney U.

[c]P adjust.: p values adjusted according to Benjamini-Hochberg.

[d] s: synonymous, son-s: non-synonymous.

[e]Amino acid change.

[f]Receptor-binding motif.

[g]Structural changes potentially impacting receptor- and antibody binding; the corresponding aa changes are indicated.

[h]This position was the only site more commonly A→G mutated in samples with high viral load.

Supplementary Table 5. Primers for sequencing.

Primer target	Genomic positions[a]	Primer sequence including Ion Torrent adapters[b]
Orf1ab RdRp (amplicon 1)	15341-15592	
1F_CT		GCCAGGTTCCAGTCACGACCCTCACTTGTTCTTGCTCGCA
1R_P1		CTCTCTATGGGCAGTCGGTGATTATCGGCAATTTTGTTACCATCAGA
Orf1ab exonuclease (amplicon 2)	18188-18414	
2F_CT		GCCAGGTTCCAGTCACGACCCTATAGAAGACTCATCTCTATGA
2R_P1		CTCTCTATGGGCAGTCGGTGATAACATAACCTGTAGGTACAGCAACT
Spike (amplicon 3)	22854-23768	
F1_CT		GCCAGGTTCCAGTCACGACTGCGTTATAGCTTGGAATTCT
R1_P1		CTCTCTATGGGCAGTCGGTGATGCTGGTGCATGTAGAAGTTCA
F2_CT		GCCAGGTTCCAGTCACGACGGTTACCAACCATACAGAGTAG
R2_P1		CTCTCTATGGGCAGTCGGTGATGTGTTATAACACTGACACCACCA
F3_CT		GCCAGGTTCCAGTCACGACACTTGAGATTCTTGACATTACACCAT
R3_P1		CTCTCTATGGGCAGTCGGTGATCACCAATAGGTATGTCACACTCAT
F4_CT		GCCAGGTTCCAGTCACGACGTTTAATAGGGGCTGAACATGTCA
R4_P1		CTCTCTATGGGCAGTCGGTGATCTGATGTCTTGGTCATAGACACTG
E (amplicon 4)	26222-26474	
E1F_CT		GCCAGGTTCCAGTCACGACCACAAGCTGATGAGTACGAACT
E2R_P1		CTCTCTATGGGCAGTCGGTGATGTTTAGACCAGAAGATCAGGAACT

[a]Region covered by the amplicon, including primer target regions that were trimmed off for bioinformatical and statistical analyses. [b] Ion Torrent adapter sequences are underlined.

Supplementary material and methods

Description of the patients

The samples were collected during March 2020 from 69 patients, who were admitted to hospitals in the Västra Götaland region in Sweden and which were tested as part of routine diagnostics. 36 patients had mild symptoms, 10 moderate symptoms, which was defined by need of supplementary oxygen, 13 were admitted to ICU and 10 patients deceased (Table 1). The patients were 38 males and 31 females, being between 1 and 97 years old. 16 patients had fever at admission, defined as a body temperature ≥ 38ºC, 11 had normal temperature, while for 42 patients these data were missing. CRP was documented for 36 patients (1 – 480, median 103 mg/L) and the time between sampling and onset of symptoms was recorded for 31 patients (1 – 16 d, median 4.5 d). 21 patients had COVID-19-associated co-morbidities, which are listed in material and methods. Ct values at time of admission were recorded for all patients, and for some patients, multiple samples were available, allowing us to analyse a total of 93 samples. Second and third samples were taken 1 – 26 d after the first (median 4 d, average 5.5 d).

SARS-CoV-2 RT-qPCR

RT-qPCR was performed for on a 7300 Fast Real-Time PCR system (Applied Biosystems). The reaction was performed in a 25 μL reaction mixture containing 5 μL sample, 1 x Reaction Mix (Invitrogen), 20 U RNaseOUT (Invitrogen), 0.5 μL SuperScript® III/platinum® Taq Mix (Invitrogen), and 0.3 μM of each primer, and 0.2 μM of probe. The qPCR started with reverse transcription at 46 °C for 30 min followed 95 °C for 10 min and 45 cycles of 95 °C for 15 sec and 56 °C 1 min. The sequence of the forward primer was GTC ATG TGT GGC GGT TCA CT, the reverse primer CAA CAC TAT TAG CAT AAG CAG TTG T and the probe CAG GTG GAA CCT CAT CAG GAG ATG C. A plasmid containing the target region of the PCR (pEX-A128-Wuhan nCov 2, Eurofins genomics) was used to determine the sensitivity of assay, which is 2 copies at Ct 40.

RNA extraction: RNA was extracted from nasopharyngeal samples using total nucleic acid extraction kit on the MagnaPure LC 2.0 instrument (Roche Life Sciences).

cDNA generation: RNA was reversely transcribed using the RevertAid First Strand cDNA synthesis kit (Thermo Fisher Scientific, Waltham, MA, USA). Per 5 μL RNA sample, 1 μL of 100 μM random hexamer primers and 6 μL of nuclease-free water were added to the reaction mixture. The reaction mixture was incubated for 5 min at 65°C in a thermal cycler to remove secondary structures. The samples were put on ice, and 2 μL of 5x Reaction Buffer, 20 U of RiboLock RNase Inhibitor, 2 μL of 10 mM dNTP mix, and 200 U of RevertAid M-MuLV RT were added to the reaction for a final volume of 20 μL. The cDNA synthesis reaction mixtures were incubated in a Veriti™ 96-Well thermal cycler using the following program: i) 25°C for 5 min, ii) 50°C for 60 min, and iii) 85°C for 5 min.

Pre-NGS amplification: PCR was performed on cDNA using fusion primers targeting different regions of the SARS-CoV-2 genome. Fusion primers targeting certain SARS-CoV-2 regions also contained adapters for Ion Torrent sequencing, and those were attached on amplicons in the same PCR. Primer sequences are found in supplementary. Barcoding PCR, to enable pooling of samples, was performed in the same reaction. Concentrations of barcoded DNA libraries from all samples were measured using a Qubit (Thermo Fisher) device and the dsDNA kit (Thermo Fisher).

Reaction mixtures consisted of 5 μL of cDNA, 5 μL of 1 μM barcode, 15 μL of 2x Platinum Hot-Start PCR Master Mix (Invitrogen), 0.5 μL of 10 μM reverse primer, 1.7 μL of 1 μM forward primer, and 2.8 μL SuperQ water for a total volume of 30 μL. The samples were incubated in a thermal cycler with the following program: i) 98°C for 30 sec, ii) 28°C for 15 sec, iii) 55°C for 15 sec, iv) 72°C for 3 min, and v) 72°C for 5 min; steps ii-iv were cycled 35 times. Samples with Ct values >27 were first subjected to a nested PCR step prior to NGS fusion. For nested PCR, the program and reaction mixture described above were used excluding

the barcodes which were substituted with SuperQ water. NGS fusion was then performed excluding the forward primers which were substituted with SuperQ water in the reaction mixture, and steps ii-iv were cycled 15 times instead of 35.

Pooling and Deep sequencing: Samples were diluted with different volumes of nuclease-free water, creating equal DNA concentrations of all samples before pooling and PCR clean-up using Sera-Mag Select (Cytiva). The pooled libraries were loaded onto Ion 520 Chip and sequencing was performed on the S5 System (XL, Prime; Thermo Fisher) according to the manufacturer's protocol for 200 bp read length. Ion Torrent deep sequencing data from all samples may be downloaded from NCBI (BioProject accession PRJNA772935 https://www.ncbi.nlm.nih.gov).

SI References

1. J. F. Piccirillo, *et al.*, The changing prevalence of comorbidity across the age spectrum. *Crit Rev Oncol Hematol* **67**, 124–132 (2008).

2. I. M. Rea, *et al.*, Age and Age-Related Diseases: Role of Inflammation Triggers and Cytokines. *Front Immunol* **9**, 586 (2018).

3. C. M. Petrilli, *et al.*, Factors associated with hospital admission and critical illness among 5279 people with coronavirus disease 2019 in New York City: prospective cohort study. *BMJ* **369**, m1966 (2020).

4. E. Meffre, A. Iwasaki, Interferon deficiency can lead to severe COVID. *Nature* **587**, 374–376 (2020).

5. A. Khera, *et al.*, Race and gender differences in C-reactive protein levels. *J Am Coll Cardiol* **46**, 464–469 (2005).
